# Artemisinins inhibited the synergistic cariogenic effect between *Candida albicans* and *Streptococcus mutans*

**DOI:** 10.3389/fcimb.2026.1950360

**Published:** 2026-09-08

**Authors:** Jingzhi Zhou, Ziyi Zhou, Jiawei Shen, Yifan Lin, Lingrui Lei, Lichen Gou, Zirui Meng, Yujie Zhou, Binyou Liao, Lei Cheng, Xuedong Zhou, Biao Ren

**Affiliations:** 1 State Key Laboratory of Oral Diseases, National Center for Stomatology, National Clinical Research Center for Oral Diseases, West China School of Stomatology, Sichuan University, Chengdu, Sichuan, China; 2 West China Lecheng Hospital, Sichuan University, Boao, Hainan, China; 3 Department of Clinical Laboratory, West China School of Stomatology, Sichuan University, Chengdu, Sichuan, China; 4 Guangdong Provincial Key Laboratory of Stomatology, Guanghua School of Stomatology, Sun Yat-Sen University, Guangzhou, China; 5 Department of Cariology and Endodontics, West China School of Stomatology, Sichuan University, Chengdu, Sichuan, China

**Keywords:** artemisinin compounds, *Candida albicans*, dental caries, *Streptococcus mutans*, synergistic cariogenic

## Abstract

**Background:**

Dental caries, a globally prevalent disease, burdens health and economies. Targeting *Candida albicans-Streptococcus mutans* synergism aids managing severe early childhood caries and root caries. Artemisinin, a natural antimalarial, inhibits *C. albicans* virulence via hyphal and biofilm suppression, promising to disrupt this cross-kingdom cariogenic partnership.

**Objective:**

This study evaluated the inhibitory effects of seven common artemisinin compounds on the synergistic cariogenic effect of *C. albicans-S. mutans* biofilm, in order to expand the application of artemisinin compounds in the field of caries prevention.

**Methods:**

Hyphal induction, biofilm formation, and CFU assays evaluated the inhibitory efficacy of the compounds against *C. albicans* and *S. mutans*. RT-qPCR examinations analyzed the expression of key virulence genes in both species. The anti-caries efficacy of artemisinin compounds was further validated in a rat caries model, demonstrated by quantitative analysis of microbial load in dental plaque and Keyes scoring for molar carious lesions.

**Results:**

All seven artemisinin derivatives effectively inhibited hyphal formation in *C. albicans*. Artemisinic acid and dihydroartemisinic acid additionally exhibited direct antibacterial activity against *S. mutans*, significantly suppressing dual-species biofilm formation, EPS synthesis, and acid production. All compounds downregulated key virulence genes in both pathogens. *In vivo* studies confirmed that artemisinic acid and dihydroartemisinic acid reduced microbial colonization and caries severity, while other derivatives primarily exerted anticaries effects by inhibiting *C. albicans* colonization.

**Conclusion:**

Artemisinin compounds can reduce the synergistic cariogenic virulence of *C. albicans* and *S. mutans* by inhibiting the interaction between *C. albicans* and *S. mutans*.

## 1 Introduction

Dental caries is a chronic and destructive disease of dental hard tissues in which microbial infection and biofilm-mediated metabolic activity play central pathogenic roles. It is the most prevalent oral disease worldwide and may adversely affect mastication, nutrition, speech, quality of life, and general health (Collaborators, 2025; Wen et al., 2022; Pitts et al., 2017). In addition, untreated dental caries and associated odontogenic infections may aggravate or contribute to systemic conditions. Therefore, dental caries has long been regarded as one of the major diseases requiring prioritized prevention and control. It remains a substantial public health burden across different age groups and that more effective preventive strategies are urgently needed (Collaborators, 2025; Wen et al., 2022).

Dental plaque biofilm is the principal initiating factor in the development of dental caries (Pitts et al., 2017; Bowen et al., 2018). Within this complex microbial community, *S. mutans* is widely recognized as one of the major cariogenic bacteria because of its strong capacities for adhesion, extracellular polysaccharide (EPS) production, acid generation, and acid tolerance (Lemos et al., 2019; Ellepola et al., 2019; Bowen and Koo, 2011). In the presence of dietary sucrose, *S. mutans* secretes glucosyltransferases that convert sucrose into EPS (Bowen and Koo, 2011; Paes Leme et al., 2006). These polymers promote bacterial aggregation and stable attachment to the tooth surface, thereby facilitating the establishment and maturation of dental plaque biofilms (Bowen and Koo, 2011; Koo et al., 2013; Klein et al., 2015). At the same time, *S. mutans* metabolizes fermentable carbohydrates and continuously produces organic acids (Lemos et al., 2019). Repeated or prolonged acid production creates a persistently acidic microenvironment at the biofilm-tooth interface (Bowen et al., 2018; Klein et al., 2015). When the local pH falls below the critical threshold for mineral stability, enamel or dentin demineralization exceeds remineralization, eventually resulting in structural breakdown and cavitation (Featherstone, 2008; Takahashi and Nyvad, 2008).

Although dental caries has traditionally been considered a predominantly bacterial disease, accumulating evidence indicates that oral fungi also participate in its initiation and progression (Man et al., 2025; Du et al., 2021). Numerous clinical studies have reported a high rate of co-detection of *C. albicans* and *S. mutans* in patients with early childhood caries (ECC) and root caries (Xiao et al., 2016; Garcia et al., 2021, 2026). Moreover, the abundance or detection frequency of these two microorganisms is positively associated with caries severity (Xiao et al., 2016). *C. albicans* is the predominant opportunistic pathogenic fungus in human oral cavity. It commonly colonizes oral mucosal surfaces and may cause oral candidiasis under conditions of impaired host defense or ecological imbalance (Vila et al., 2020). One of the most important virulence characteristics of *C. albicans* is its ability to undergo morphological transition from budding yeast cells to elongated hyphae (Kumamoto and Vinces, 2005; Chen et al., 2020). Hyphal development contributes to tissue adhesion, invasion, biofilm maturation, epithelial damage, and resistance to host immune clearance (Hoyer and Cota, 2016; Ponde et al., 2021; Nobile and Johnson, 2015).

*C. albicans* exhibits relatively weak cariogenicity when present alone (Sampaio et al., 2019; Falsetta et al., 2014). However, it can markedly enhance the development of ECC and root caries through cross-kingdom interactions with *S. mutans* (Du et al., 2021). A key mechanism underlying this synergistic relationship involves glucosyltransferase B (GtfB), an extracellular enzyme secreted by *S. mutans* (Gregoire et al., 2011; Hwang et al., 2017).In sucrose-rich environments, *S. mutans*-derived GtfB and GtfC synthesize extracellular glucans, a component of the EPS, which assemble into an adhesive matrix on tooth surfaces and around bacterial microcolonies (Falsetta et al., 2014; Xiao et al., 2012). This EPS-rich matrix can surround both bacterial and fungal cells, enhance biofilm cohesion, impede neutralization, and promote the local retention of acids, thereby facilitating the establishment of localized acidic microenvironments (Falsetta et al., 2014; Hwang et al., 2016). Extracellular products derived from dual-species biofilms can stimulate *gtfB/C* expression and Gtf activity in *S. mutans*, whereas glucose and fructose generated through *S. mutans*-mediated sucrose metabolism can be utilized by *C. albicans* (Kim et al., 2017; Ellepola et al., 2019).

*C. albicans* can also promote the expression of *gtf* genes in *S. mutans*, further increasing EPS production (Kim et al., 2017; Falsetta et al., 2014). The locally generated EPS enhances coaggregation between *C. albicans* and *S. mutans* and improves the adhesion of both microorganisms to tooth surfaces (Gregoire et al., 2011; Hwang et al., 2017). Adhesin-mediated interactions involving *S. mutans* antigen I/II and the *C. albicans* Als1 and Hwp1 may further reinforce direct interspecies attachment and dual-species biofilm formation (Yang et al., 2018; Martorano-Fernandes et al., 2023). This EPS-rich matrix also increases biofilm biomass, mechanical stability, and resistance to environmental stresses (Kim et al., 2018; Hwang et al., 2017). Conversely, *S. mutans* can enhance the adhesion of *C. albicans* and promote the expression of fungal genes involved in hyphal development and biofilm formation (Ellepola et al., 2017). These bidirectional interactions lead to the formation of a highly organized and more virulent dual-species biofilm (Falsetta et al., 2014). Compared with single-species biofilms, the cross-kingdom biofilm exhibits greater biomass accumulation, acidogenicity, acid tolerance, and cariogenic potential (Lobo et al., 2019; Ellepola et al., 2019). Therefore, disrupting the synergistic interactions between *C. albicans* and *S. mutans* could be an important strategy for the prevention and treatment of ECC and root caries.

Pharmacological intervention is an important component of non-invasive or minimally invasive management for early carious lesions (Slayton et al., 2018). Fluoride remains the most commonly used anticaries agent, and its effectiveness in promoting remineralization and reducing enamel demineralization has been well established (Featherstone, 1999). However, inappropriate or excessive fluoride exposure may increase the risk of dental fluorosis, and prolonged microbial exposure to fluoride-containing products may contribute to the selection of fluoride-tolerant strains (Wong et al., 2024). Antimicrobial agents have also been investigated for caries prevention, but their long-term application may induce microbial resistance and substantially disturb the ecological balance of the oral microbiome (Bartsch et al., 2024). Therefore, the identification of safer and more effective anti-caries compounds has become the main focus of current research.

Natural products have attracted increasing attention because of their diverse chemical structures, broad biological activities, wide availability, and generally favorable safety profiles (Chen et al., 2020). Natural substances derived from *Magnolia officinalis*, *Galla chinensis*, honeycomb, tea polyphenols, and other medicinal materials have been reported to suppress cariogenic microorganisms, inhibit virulence-factor production, reduce biofilm formation, or promote the ecological control of dental plaque (Kashi et al., 2025). Nevertheless, most studies of natural anticaries agents have focused on individual bacterial species or single-species biofilms. Effective compounds capable of specifically disrupting the fungal-bacterial interaction between *C. albicans* and *S. mutans* remain limited.

Artemisinin is a naturally occurring antimalarial sesquiterpene lactone isolated from *Artemisia annua* L., a medicinal herb widely used in traditional Chinese medicine (Miller and Su, 2011). To improve its pharmacological properties and clinical applicability, several artemisinin derivatives have been developed. In recent years, artemisinin and its derivatives have been shown to possess biological activities beyond their well-established antimalarial effects. Artemisinin compounds originate from the same biosynthetic pathway and structural framework, but exhibit considerable variation in several respects. In order to cover the chemical diversity, we chose seven principal artemisinin compounds--artemisinin and six related compounds, including dihydroartemisinin, artesunate, artemether, arteether, artemisinic acid and dihydroartemisinic acid. Artemisinin was included as the natural parent scaffold, whereas dihydroartemisinin, the reduced lactol derivative of artemisinin, represents a central active compound and the structural precursor of several clinically relevant derivatives (O'Neill and Posner, 2004; Parapini et al., 2015; Jansen, 2010). Artesunate, artemether, and arteether represent relatively hydrophilic ester derivative and two lipophilic ether derivatives (de Vries and Dien, 1996; Jansen, 2010). Artemisinic acid is a biosynthetically related branch metabolite and an established precursor for semi-synthetic artemisinin production, whereas dihydroartemisinic acid is a late-stage natural biosynthetic precursor of artemisinin; neither compound contains the characteristic endoperoxide bridge (Brown, 2010; Westfall et al., 2012; Paddon et al., 2013). These compounds enabled a focused comparison across biosynthetic position, polarity and endoperoxide status.

Previous studies have demonstrated that artemisinin and a precursor of artemisinin, exert bacteriostatic activity against *S. mutans*, whereas several other artemisinin-related compounds display limited direct inhibition of bacterial growth (Appalasamy et al., 2014). Early investigations also suggested that artemisinin had only weak activity against *C. albicans*. However, more recent evidence indicates that artemisinin compounds may have considerable potential for controlling *C. albicans*-associated infection (Galal et al., 2005). Our group previously showed that artemisinin did not significantly inhibit the yeast growth of *C. albicans*, it could act synergistically with antifungal agents such as amphotericin B, reduced oral lesions, fungal burden, epithelial infection, and inflammation (Zhu et al., 2021). More importantly, artemisinin compounds have been shown to suppress the yeast-to-hypha transition of *C. albicans*, without substantially suppressing fungal growth. Arteether showed particularly strong antihyphal activity and decreased fungal burden and hyphal invasion *in vivo* (Liang et al., 2023).

In this study, we investigated the inhibitory effects of seven artemisinin-related compounds on the synergistic cariogenicity of *C. albicans* and *S. mutans* for the first time. The effects of these compounds on the growth, biofilm formation, EPS production, acidogenicity, acid tolerance, and other cariogenic virulence characteristics of dual-species biofilms were first evaluated *in vitro*. RT-qPCR was subsequently performed to determine their effects on the expression of virulence-associated genes in both *C. albicans* and *S. mutans*. And a rat caries model was established to assess the *in vivo* anticaries efficacy of the selected compounds. Our findings identify artemisinin compounds as promising candidates for the prevention of cross-kingdom biofilm-associated dental caries and provide a new basis for expanding the application of traditional Chinese medicine-derived compounds in oral healthcare.

## 2 Materials and methods

### 2.1 Chemicals

Artemisinin, dihydroartemisinin, artesunate, and artemether were purchased from J&K Scientific (Beijing, China). Arteether, artemisinic acid, and dihydroartemisinic acid were obtained from Solarbio (Beijing, China). Dimethyl sulfoxide (DMSO) was acquired from MP Biomedicals (Irvine, CA, USA). All compounds were dissolved in DMSO and stored at -20 °C. In all experiments, the final DMSO concentration was maintained at 0.1% (v/v), and all treatment and control groups contained an equivalent concentration of DMSO.

### 2.2 Strains

*S. mutans* UA159 and *C. albicans* SC5314 (ATCC MYA-2876) were obtained from the State Key Laboratory of Oral Diseases. Before each experiment, *S. mutans* was cultured overnight in brain-heart infusion (BHI) medium, whereas *C. albicans* was cultured in yeast extract-peptone-dextrose (YPD) medium at 35 °C under appropriate growth conditions.

### 2.3 Antimicrobial susceptibility testing

*C. albicans* was suspended in RPMI 1640 medium at a final concentration of 2.5 × 10^4^ colony-forming units (CFU)/mL, while *S. mutans* was adjusted to 5 × 10^5^ CFU/mL in BHI medium. Each test compound was prepared by twofold serial dilution to obtain final concentrations of 400, 200, 100, 50, 25, 12.5, 6.25, and 3.12 mg/L. Wells containing an equivalent volume of DMSO without the test compound served as the negative control. After incubation for 24 h under appropriate growth conditions, microbial growth was assessed visually, and the minimum inhibitory concentration (MIC) was defined as the lowest compound concentration that completely prevented visible growth (Zhou et al., 2018; Lu et al., 2019).

### 2.4 Hyphal formation measurement

*C. albicans* in yeast nitrogen base broth (YNBB) to a final concentration of 5 × 10^5^ CFU/mL. The fungal suspensions were treated with the indicated artemisinin compounds at final concentrations of 50, 100, 200, or 400 mg/L. A vehicle-treated group containing an equivalent concentration of DMSO was included as the negative control. After incubation at 37 °C, 5% CO_2_ for 24 h, fungal morphology was imaged using a Cytation 5 multimode imaging system (BioTek, Winooski, VT, USA), and hyphal development was evaluated (Sztajer et al., 2014).

### 2.5 Crystal violet staining assay

S. *mutans* (2×10^6^ CFU/mL) and *C. albicans* (2×10^4^ CFU/mL) in YNBB medium were treated with artemisinin compounds at 400, 200, 100, 50 mg/L at 37 °C under 5% CO_2_ for 24 h, after washing and fixing with anhydrous methanol, 0.1% CV solution was added and incubated for 15 min for staining. After washing, anhydrous ethanol was added and shaken for 20 min. The CV solutions were then transferred to a new 96-well plate. Absorbance was measured at 595 nm with a microplate reader (SpectraMax ID3, Thermo Fisher, USA) (Mataraci and Dosler, 2012).

### 2.6 Count of biofilm CFU

After incubation for 24 h, single- and dual-species biofilms of *S. mutans* and *C. albicans* formed in 96-well plates were gently washed with sterile phosphate-buffered saline (PBS). The biofilms attached to the well surfaces were scraped, resuspended in PBS, and sonicated for 5 min to disperse microbial aggregates. Suspensions were then plated onto CHROMagar plates and mitis salivarius-bacitracin (MSB) agar plates for selective counting of *C. albicans* and *S. mutans* colonies, respectively. CHROMagar *Candida* plates were incubated aerobically at 37 °C for 48 h, and smooth green colonies were presumptively identified as *C. albicans*. MSB agar plates were incubated at 37 °C in 5% CO_2_ for 48 h, and small, raised, opaque, blue colonies with a frosted-glass appearance and firm adherence to the agar surface were identified as *S. mutans*.

### 2.7 Acid production assay of biofilms

*S. mutans* (2 × 10^6^ CFU/mL) and *C. albicans* (2 × 10^4^ CFU/mL) were co-cultured in YNBB medium containing artemisinin compounds at final concentrations of 50, 100, 200, or 400 mg/L. After incubation at 37 °C, 5% CO_2_ for 24 h, the supernatants were removed and the biofilms were washed. New YNBB medium was then added, followed by a further 24h incubation. The pH of the resulting culture medium was measured using a pH meter.

### 2.8 Scanning electron microscopy

Cell climbing slices were placed into the wells, and biofilms were prepared following the aforementioned method and cultured for 24 h. After discarding the supernatant and washing, glutaraldehyde solution was added for overnight fixation. Subsequently, the samples were subjected to gradient dehydration using 50%, 60%, 70%, 80%, 90%, 95%, and absolute ethanol. After drying and gold sputtering, images were acquired using a SEM (JSM-7500F; Jeol Ltd, Tokyo, Japan) at a magnification of 5000× (Han et al., 2017).

### 2.9 Quantification of water-insoluble EPS

The sulfuric acid-anthrone assay was used to evaluate the effects of artemisinin compounds on water-insoluble EPS in dual-species biofilms. Anthrone powder was added to concentrated sulfuric acid to prepare an anthrone solution with a mass concentration of 0.2%. Biofilms were established in 96-well plates according to the method described previously, after 24 h, the biofilms were scraped off, resuspended in PBS, and centrifuged to discard the supernatant. Subsequently, 0.2% anthrone solution was added to the pellet, followed by incubation in a water bath at 95°C for 10 min. The liquid was aspirated and transferred to a 96-well plate, and the absorbance value was measured at 625 nm using a microplate reader (Wang et al., 2020).

### 2.10 EPS staining of biofilms

Biofilms were cultured in confocal dishes at concentrations of 400 and 200 mg/L of artemisinin compounds according to the method described previously. 1 μM alexa Fluor^®^ 647 (Invitrogen, CA, USA) was added to the culture medium, and the samples were incubated in the dark for 24 h. The supernatant was aspirated and discarded, after which 2 μM of the nucleic acid dye SYTO9 (Invitrogen, CA, USA) was added for 15 min. Subsequently, the dyes were washed off, and the stained samples were observed under a laser scanning confocal microscope (FV1000, Olympus, Tokyo, Japan).

### 2.11 RT-qPCR

After 24 h of biofilm cultivation, the supernatant was removed, and the biofilms were scraped off with a scraper. Then, 1 mL of Trizol lysis reagent was added, the collected biofilm samples were transferred to lysis tubes for cell disruption. Total RNA was extracted according to the method described previously, and subsequently reverse transcribed into cDNA. The cDNA was analyzed by RT-qPCR, and gene expression was detected using a real-time PCR system (Roche, Basel, Switzerland). All the primers used are listed in **Supplementary Table 1**.

### 2.12 Rat dental caries model and treatment

A total of 168 female Sprague-Dawley rats (21 days old) were used. Rats were randomly divided into three infection groups from 26–30 d: mono-infection with *C. albicans*, mono-infection with *S. mutans*, or co-infection with both. Oral inoculation was performed once daily on postnatal days 26-30. During inoculation, anesthesia was induced with 3.5% isoflurane and maintained with 2% isoflurane. For mono-infection, each rat received 500 μL of either *C. albicans* at 5 × 10^7^ CFU/mL or *S. mutans* at 5 × 10^9^ CFU/mL. For co-infection, equal volumes of the *C. albicans* suspension (5 × 10^7^ CFU/mL) and the *S. mutans* suspension (5 × 10^9^ CFU/mL) were mixed; each rat received 500 μL of the mixed inoculum. Throughout the experiment, all rats were fed a high-sucrose cariogenic diet (Diet 2000) and provided with 5% sucrose water (Keyes, 1958; Falsetta et al., 2014). Each infection condition was further divided into eight treatment groups: seven artemisinin-related compound groups and one vehicle-control group, resulting in 24 groups (n = 7). From day 31, each compound was administered at 400 mg/L. Rats received twice-daily topical oral applications of their assigned solutions for 5 weeks, 2 mL of the assigned solution was prepared for each rat. All compound and control solutions contained 0.1% (v/v) DMSO. A sterile cotton swab was soaked in the assigned solution and used to thoroughly swab the molar surfaces and oral mucosa. The remaining solution was then introduced into the oral cavity using a blunt-ended syringe to rinse the oral surfaces. Food and water were withheld for 1 h after each treatment. On postnatal day 66, the rats were euthanized by gradual-fill CO_2_ inhalation. The mandibles were collected and sonicated to recover the dental biofilms, and the resulting suspensions were cultured on selective agar for colony enumeration. Carious lesions on the rat molars were examined under a stereomicroscope and scored according to the Keyes criteria. Ethical approval was received from Ethics Committee of West China Hospital of Sichuan University [Judgment’s reference no. WCHSIRB-D-2022-033].

### 2.13 Statistical analysis

Statistical analyses were conducted with GraphPad Prism 10.1.2 (GraphPad, San Diego, CA, USA). Data following a normal distribution were analyzed using t-tests or ANOVA. In all analyses, *P*-value of less than 0.05 was considered statistically significant.

## 3 Results

### 3.1 Inhibitory effects of artemisinin compounds on *C. albicans* and *S. mutans*

Seven artemisinin compounds showed no antifungal activity against *C. albicans*, with MIC values > 400 mg/L. At a high concentration (400 mg/L), artemisinic acid and dihydroartemisinic acid inhibited the growth of *S. mutans*, whereas the other compounds exhibited no significant antibacterial effect (**Table 1**).

**Table 1:** Minimum inhibitory concentrations of the seven artemisinin compounds against *C. albicans* and *S. mutans*.

Compound	MIC (mg/L)	
	*C. albicans*	*S. mutans*
Artemisinin	>400	>400
Dihydroartemisinin	>400	>400
Artesunate	>400	>400
Artemether	>400	>400
Arteether	>400	>400
Artemisinic Acid	>400	400
Dihydroartemisinic Acid	>400	400

MIC values are reported in mg/L; values > 400 mg/L indicate that growth inhibition was not detected at the highest concentration tested. MIC, minimum inhibitory concentration.

Treatment of *C. albicans* with artemisinin compounds significantly suppressed hyphal formation in a dose-dependent manner. The most pronounced inhibitory effects were observed with artemisinic acid and dihydroartemisinic acid at 400 mg/L (**Supplementary Figure 1**).

**Figure 1:**
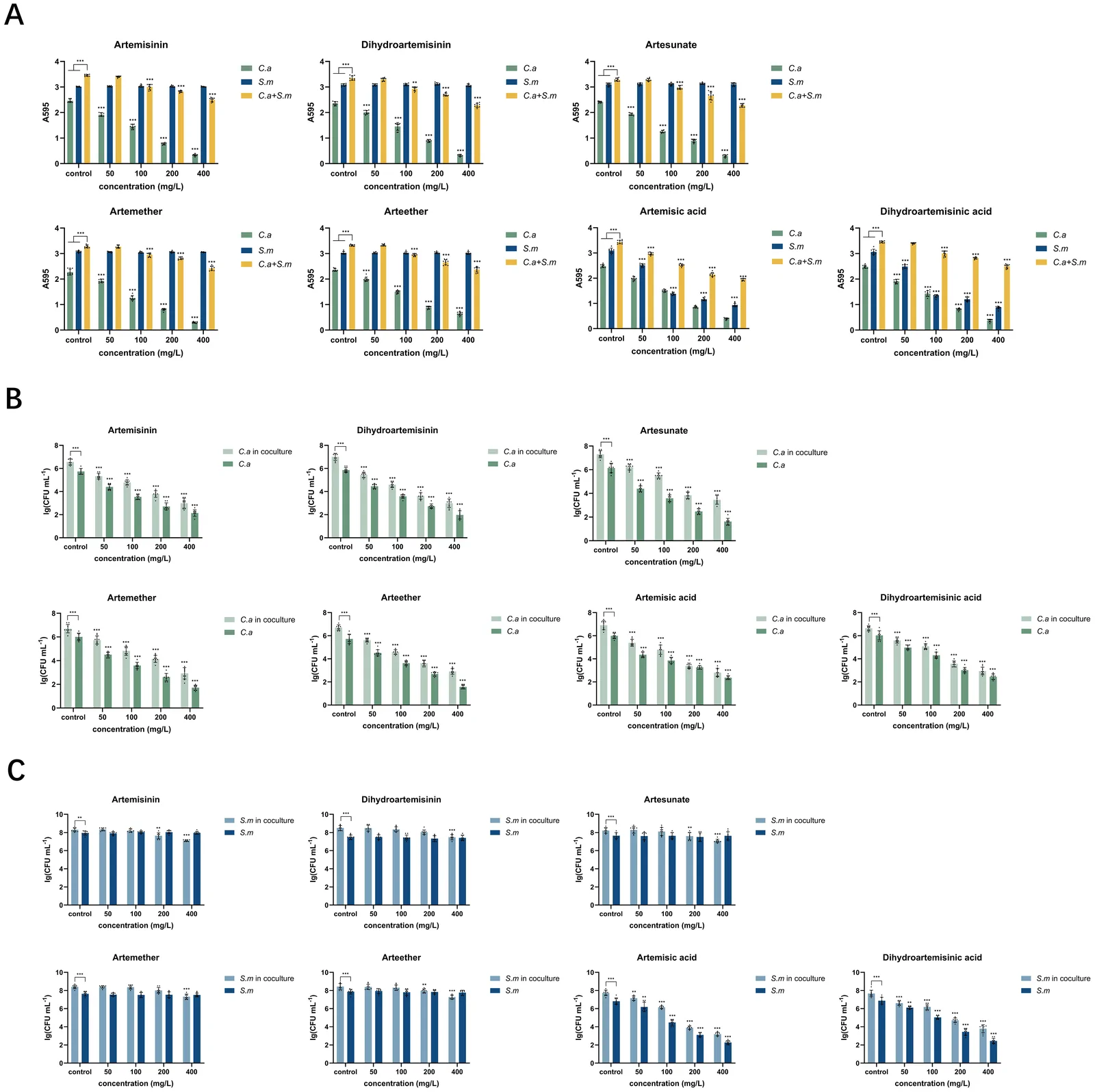
Artemisinin compounds inhibit biofilm formation and microbial accumulation in single- and dual-species biofilms. **(A)** Biofilm biomass of *C. albicans* single-species, *S. mutans* single-species, and *C. albicans-S. mutans* dual-species biofilms assessed by crystal violet staining. **(B)** Viable *C. albicans* recovered from single- and dual-species biofilms by CFU counting. **(C)** Viable *S. mutans* recovered from single- and dual-species biofilms by CFU counting. Biofilms were treated for 24 h with the indicated compound concentrations. Data are presented of three independent biological experiments (n=3). Group differences were evaluated using one-way ANOVA. CFU, colony-forming units. **p<0.01; ***p<0.001.

CV staining was employed to assess biofilm biomass. In the absence of drug treatment, co-cultivation significantly promoted biofilm formation. For *C. albicans* single-species biofilms, all seven artemisinin compounds inhibited biofilm formation in a concentration-dependent manner. For *S. mutans* single-species biofilms, artemisinic acid and dihydroartemisinic acid effectively suppressed biofilm biomass, whereas no obvious inhibitory effects were observed for the other compounds. In dual-species biofilms, all compounds reduced biofilm formation, artemisinic acid and dihydroartemisinic acid demonstrated the most significant inhibitory effects (**Figure 1A**).

CFU counting was conducted to quantify viable microorganisms within the biofilms. Co-cultivation mutually promoted the growth of the two species. All seven artemisinin compounds reduced the colonization level of *C. albicans* in both single-species and dual-species biofilms (**Figure 1B**). In contrast, only artemisinic acid and dihydroartemisinic acid inhibited *S. mutans* in single-species biofilms. In dual-species biofilms, artemisinin, dihydroartemisinin, artesunate, artemether, and arteether at 400 mg/L also decreased the CFU count of *S. mutans* (**Figure 1C**).

**Figure 2:**
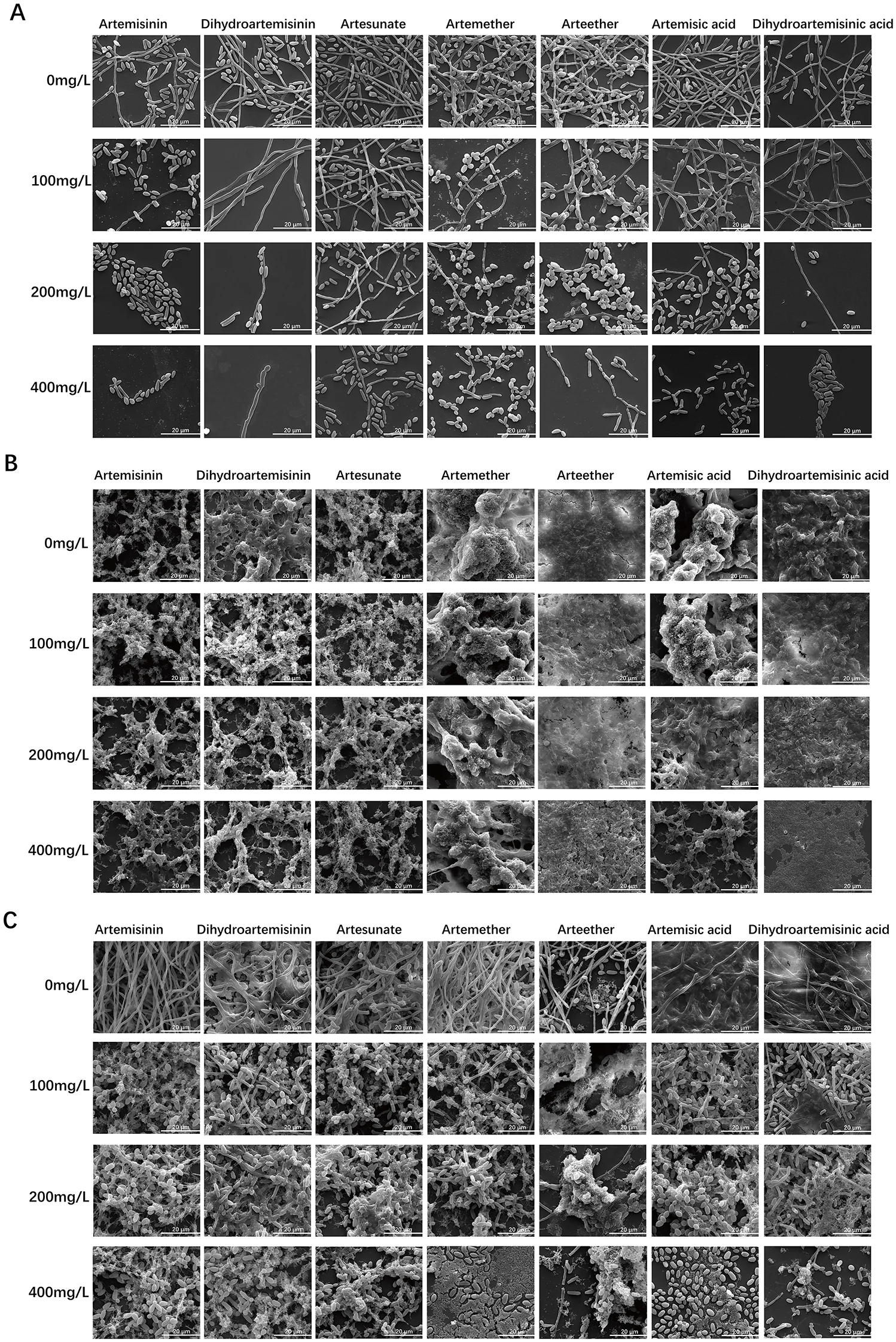
Artemisinin compounds inhibit biofilm architecture and cellular morphology in single- and dual-species biofilms. **(A)** *C. albicans* single-species biofilms, **(B)** *S. mutans* single-species biofilms, and **(C)** *C. albicans-S. mutans* dual-species biofilms after treatment with the indicated concentrations of the seven artemisinin compounds. In untreated dual-species biofilms, abundant *C. albicans* hyphae intertwine with and cover *S. mutans*, whereas treated biofilms show reduced hyphal growth and a sparser architecture. SEM, scanning electron microscopy. Images are representative of three independent biological experiments (n=3). Scale bar, 20 μm.

SEM imaging further revealed that all seven artemisinin compounds inhibited biofilm formation and hyphal transition in *C. albicans* single-species biofilms (**Figure 2A**). In *S. mutans* single-species biofilms, artemisinic acid and dihydroartemisinic acid significantly suppressed biofilm formation, whereas the other compounds had no obvious effects (**Figure 2B**). In dual-species biofilms, the control group exhibited a dense structure in which abundant *C. albicans* hyphae intertwined with and covered *S. mutans*. In contrast, artemisinin-treated biofilms were sparser and showed inhibited *C. albicans* hyphal growth (**Figure 2C**).

Collectively, these findings indicate that artemisinin compounds suppress multispecies biofilm, with artemisinic acid and dihydroartemisinic acid showing the broadest activity.

### 3.2 Artemisinin compounds reduce biofilm acidogenicity and EPS production

**Figure 3:**
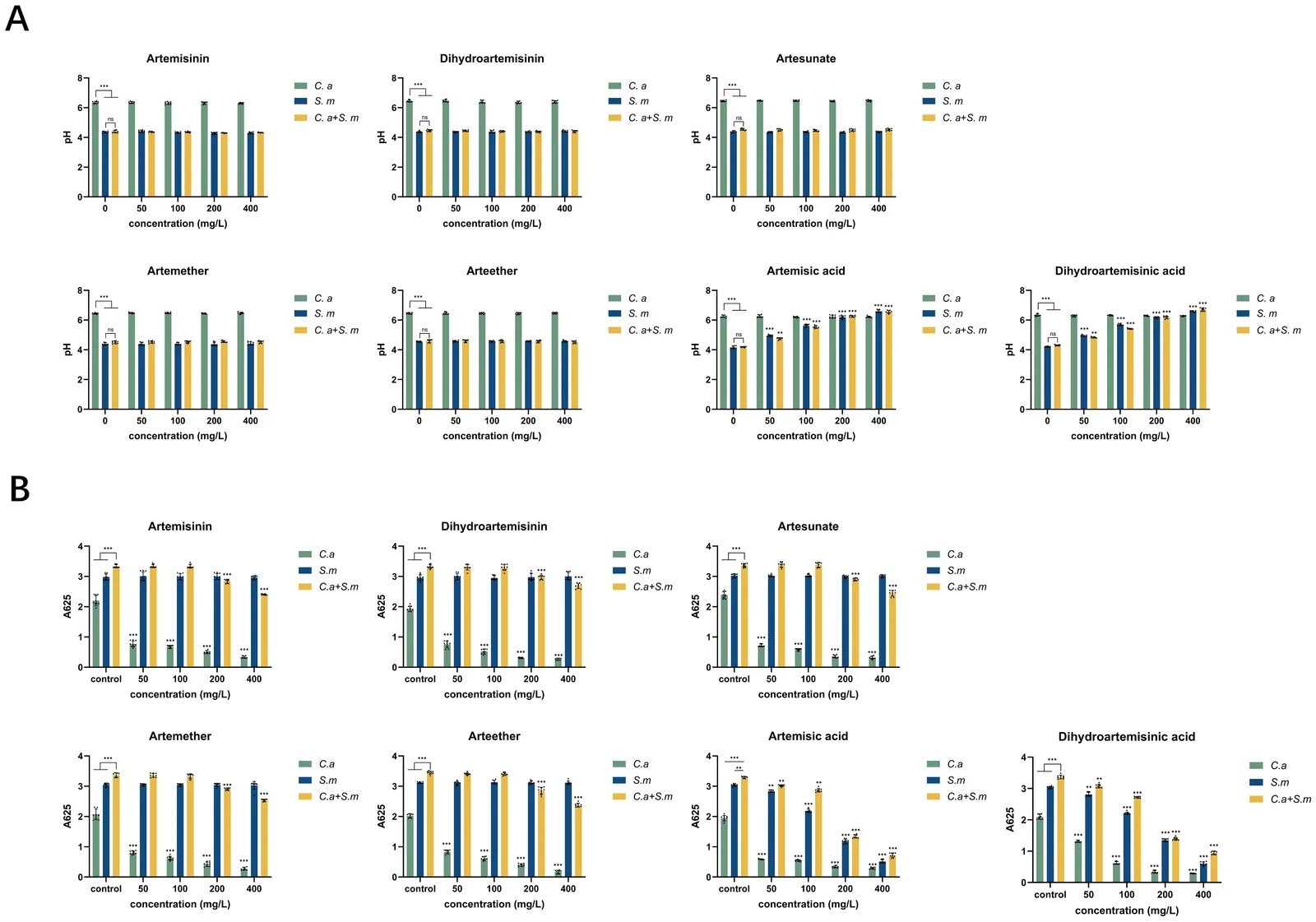
Effects of artemisinin compounds on biofilm acidogenicity and EPS production. **(A)** Supernatant pH of *C. albicans* single-species, *S. mutans* single-species, and *C. albicans-S. mutans* dual-species biofilms after treatment with the indicated concentrations. The dashed reference, where shown, denotes the critical pH of 5.5 for enamel demineralization. **(B)** Quantification of water-insoluble EPS produced by the corresponding single- and dual-species biofilms. EPS, extracellular polymeric substances. Data are presented of three independent biological experiments (n=3). Group differences were evaluated using one-way ANOVA. ns, not significant; **p<0.01; ***p<0.001.

Acid production is a key cariogenic virulence factor of *S. mutans*. The effects of artemisinin compounds on biofilm acidogenicity were evaluated by measuring the pH of the biofilm supernatant. *C. albicans* single-species biofilms exhibited only weak acid-producing ability, with no significant difference between the treated groups and the control group. In both *S. mutans* single-species and dual-species biofilms, the supernatant pH of the untreated control groups was acidic. Artemisinic acid and dihydroartemisinic acid inhibited acid production in a concentration-dependent manner at 100–400 mg/L. When the other artemisinin compounds were applied to dual-species biofilms at 400 mg/L, the pH increased slightly but remained below the critical pH of 5.5 for enamel demineralization (**Figure 3A**).

Since EPS synthesis is critical to the synergistic cariogenicity of *S. mutans* and *C. albicans*, water-insoluble EPS was quantified. All seven artemisinin compounds significantly inhibited EPS production in *C. albicans* single-species biofilms. In *S. mutans* single-species biofilms, artemisinic acid and dihydroartemisinic acid at 50–400 mg/L significantly reduced EPS yield. In dual-species biofilms, these two compounds inhibited EPS production at 50–400 mg/L, whereas artemisinin, dihydroartemisinin, artesunate, artemether, and arteether exerted significant inhibitory effects only at 200 and 400 mg/L (**Figure 3B**). Thus, artemisinic acid and dihydroartemisinic acid most strongly inhibited both acid production and EPS synthesis, while the other five compounds predominantly reduced EPS production in dual-species biofilms.

### 3.3 Artemisinin compounds disrupt biofilms architecture

To further examine biofilm architecture, 200 and 400 mg/L were selected for subsequent experiments because these concentrations showed marked inhibition of EPS production. EPS and viable microbial cells were fluorescently stained and observed by confocal laser scanning microscopy (CLSM), with viable cells shown in green and EPS shown in red.

**Figure 4:**
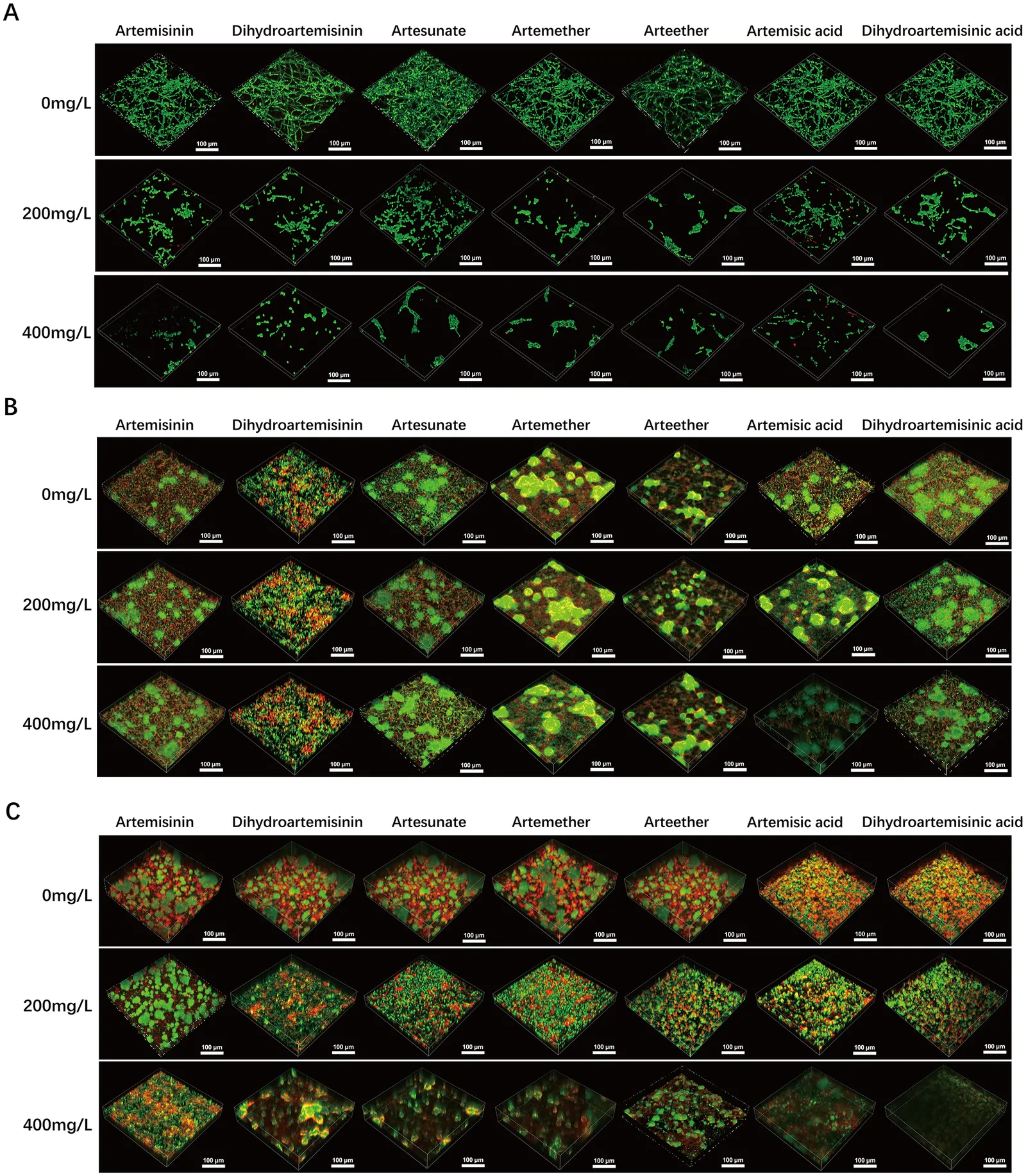
CLSM visualization of viable microbial cells and EPS within single- and dual-species biofilms. Representative images of **(A)** *C. albicans* single-species biofilms, **(B)** *S. mutans* single-species biofilms, and **(C)** *C. albicans-S. mutans* dual-species biofilms treated with 200 or 400 mg/L of the indicated artemisinin compounds. Viable microbial cells are shown in green, EPS is shown in red, and merged images illustrate their spatial distribution. CLSM, confocal laser scanning microscopy; EPS, extracellular polymeric substances. Images are representative of three independent biological experiments (n=3). Scale bar, 100 μm.

In *C. albicans* single-species biofilms, the control group contained abundant hyphae and viable cells, whereas treated biofilms showed fewer viable cells and predominantly yeast-form fungal cells (**Figure 4A**). In *S. mutans* single-species biofilms, artemisinic acid and dihydroartemisinic acid markedly reduced both EPS formation and viable-cell numbers, while no obvious changes were detected with the other compounds (**Figure 4B**). The untreated dual-species biofilm exhibited a compact structure with abundant EPS. In contrast, all treated groups showed reduced EPS, a looser biofilm architecture, and fewer viable cells, with the most pronounced inhibition observed for artemisinic acid and dihydroartemisinic acid at 400 mg/L (**Figure 4C**).

### 3.4 Artemisinin compounds inhibited virulence gene in *C. albicans* and *S. mutans*

**Figure 5:**
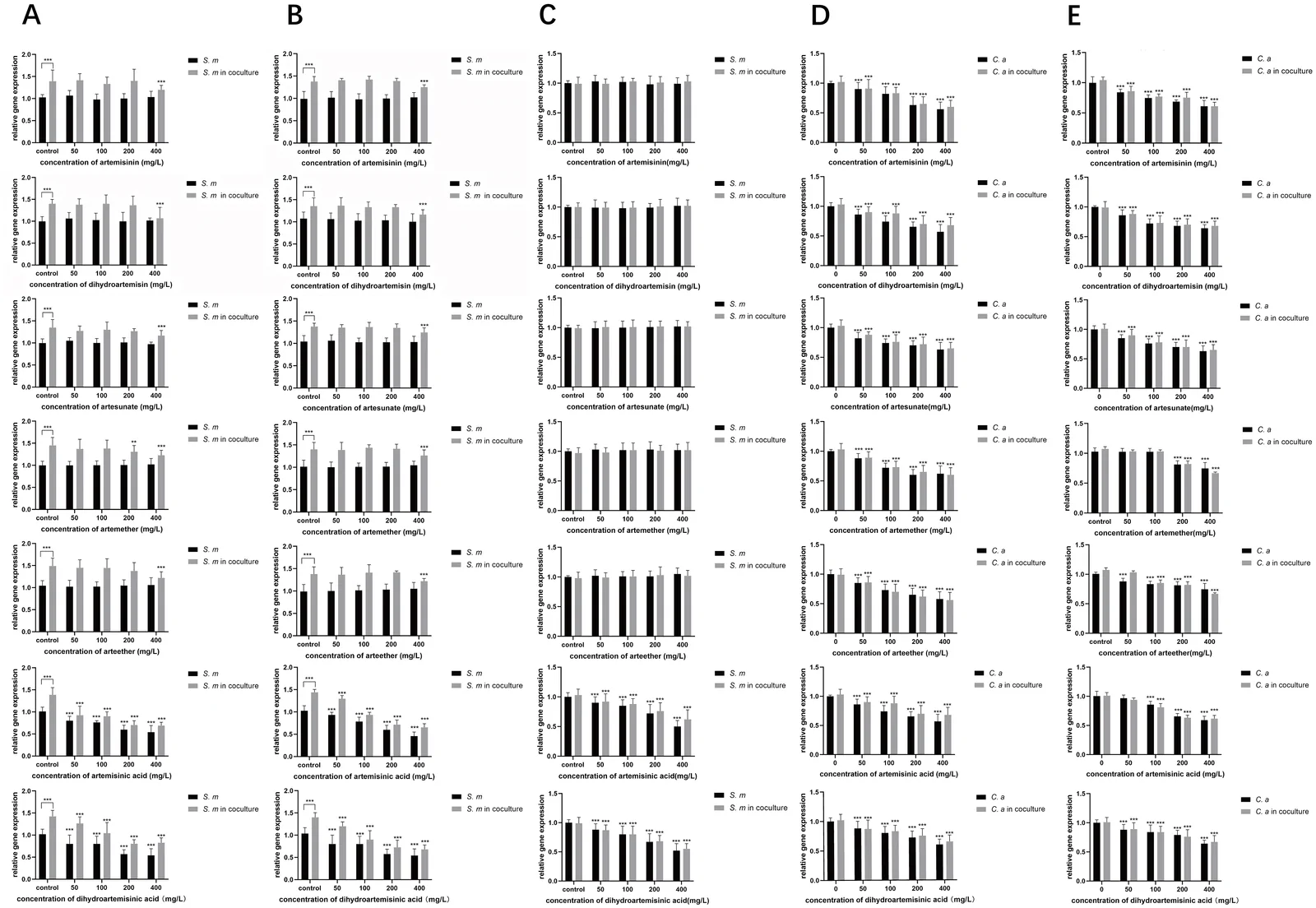
Artemisinin compounds modulate virulence-gene expression in *S. mutans* and *C. albicans* biofilms. Relative expression of the *S. mutans* genes **(A)** *gtfB*, **(B)** *gtfC*, and **(C)** *ldh*, and the *C. albicans* genes **(D)** *ALS3* and **(E)** *EFG1*, after treatment with the indicated concentrations of the seven artemisinin compounds. *gtfB* and *gtfC* encode glucosyltransferases involved in EPS synthesis; *ldh* is associated with lactic acid production; *ALS3* is associated with hyphal adhesion and interspecies aggregation; and *EFG1* regulates hyphal development. Expression is shown relative to the corresponding untreated biofilm control. Statistical annotations indicate the comparisons defined in Materials and methods. Data are presented of three independent biological experiments (n=3). Group differences were evaluated using one-way ANOVA. ***p<0.001.

The *gtfB* and *gtfC* genes are key mediators of EPS synthesis and sucrose-dependent adhesion in *S. mutans*. Their expression was upregulated in dual-species biofilms, indicating that *C. albicans* enhanced the cariogenic virulence of *S. mutans*. In single-species biofilms, only artemisinic acid and dihydroartemisinic acid inhibited *gtfB/C* expression. In dual-species biofilms, these two compounds suppressed *gtfB/C* at 50–400 mg/L, whereas artemisinin, dihydroartemisinin, artesunate, artemether, and arteether exerted inhibitory effects only at 400 mg/L (**Figures 5A, B**). Consistent with the acid-production results, only artemisinic acid and dihydroartemisinic acid downregulated *ldh*, a gene associated with lactic acid production in *S. mutans* (**Figure 5C**).

The hypha-associated gene *ALS3* is crucial for oral colonization and adhesion by *C. albicans* and contributes to its aggregation with other microorganisms, while *EFG1* is a key regulator of hyphal formation. All seven artemisinin compounds significantly downregulated *ALS3* and *EFG1* in a concentration-dependent manner (**Figures 5D, E**).

These results demonstrate that artemisinin compounds suppress virulence-gene expression in both organisms, including the EPS-synthesis genes *gtfB/C* in *S. mutans* and the hypha-associated genes *ALS3* and *EFG1* in *C. albicans*.

### 3.5 Artemisinin compounds inhibited the development of dental caries in rats

The anti-caries efficacy of the artemisinin compounds was further evaluated in rat caries models. Body weights remained comparable among all groups throughout the experimental period (**Supplementary Figure 2**).

**Figure 6:**
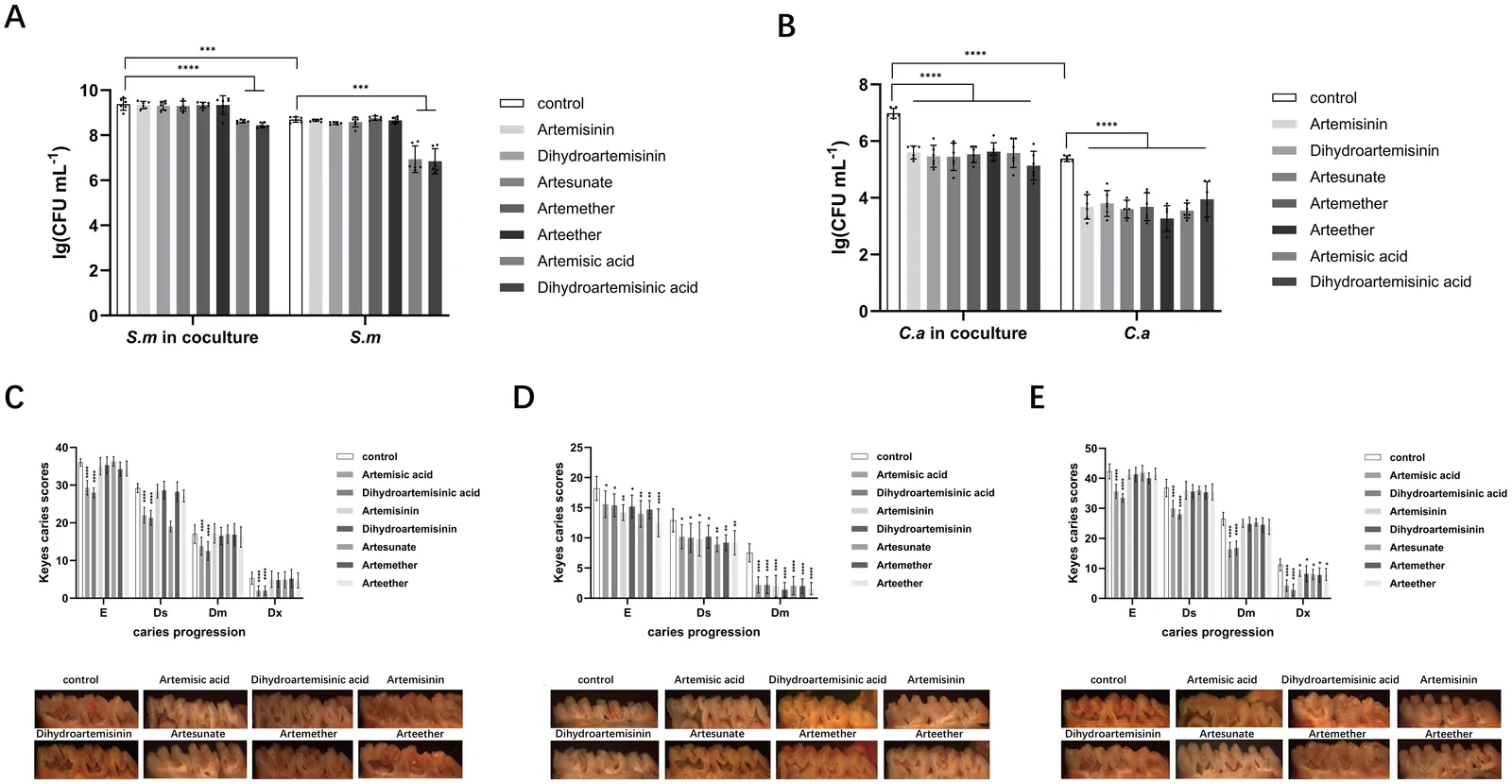
Artemisinin compounds reduce microbial colonization and caries severity in rat caries models. Viable counts of **(A)** *S. mutans* and **(B)** *C. albicans* recovered from rat dental plaque after treatment with the indicated compounds. Representative images of rat molar tooth sections and Keyes scores for sulcal caries in rats infected with **(C)** *S. mutans*, **(D)** *C. albicans*, or **(E)** both *S. mutans* and *C. albicans*. E lesions are confined to enamel; Ds lesions extend into the outer one-quarter of dentin; Dm lesions involve approximately one-quarter to three-quarters of dentin; and Dx lesions extend through more than three-quarters of dentin. Data are presented as the mean ± SD, with each rat considered an independent statistical unit (n=7 per group). Group differences were evaluated using one-way ANOVA. CFU, colony-forming units. *p<0.05; **p<0.01; ***p<0.001.

Plaque biofilms collected from rat teeth were diluted and plated for CFU counting. In both the *S. mutans* mono-infection and co-infection models, artemisinic acid and dihydroartemisinic acid significantly reduced *S. mutans* colonization, whereas no obvious changes were observed with the other compounds (**Figure 6A**). In the *C. albicans* mono-infection and co-infection models, all seven artemisinin compounds markedly decreased *C. albicans* colonization on rat tooth surfaces (**Figure 6B**).

Longitudinal sections of rat molars were examined under a stereomicroscope, and caries severity was evaluated using the Keyes scoring system. E-level lesions are confined within the enamel; Ds-level lesions involve approximately the outer one-quarter of the dentin between the enamel and the pulp chamber; Dm-level lesions affect about one-quarter to three-quarters of the dentin; and Dx-level lesions involve more than three-quarters of the dentin region. Caries developed in the *C. albicans* mono-infection, *S. mutans* mono-infection, and co-infection groups. The *C. albicans* mono-infection group exhibited the mildest lesions, whereas co-infected rats showed more severe caries than rats infected with *S. mutans* alone. In *S. mutans*-infected rats, artemisinic acid and dihydroartemisinic acid significantly reduced E-, Ds-, Dm-, and Dx-level Keyes scores, while the other compounds did not differ significantly from the control (**Figure 6C**; **Supplementary Table 2**). In *C. albicans*-infected rats, all treated groups showed lower E-, Ds-, Dm-level scores than the control group, and no Dx-level lesions were observed (**Figure 6D**; **Supplementary Table 3**). In co-infected rats, artemisinic acid and dihydroartemisinic acid reduced scores at all four lesion levels, while the other five compounds reduced scores only at the Dx-level lesions (**Figure 6E**; **Supplementary Table 4**).

Overall, all seven compounds exerted anti-caries effects in rats, with artemisinic acid and dihydroartemisinic acid showing the greatest efficacy.

## 4 Discussion

ECC and root caries represent significant public health burdens, with *C. albicans* and *S. mutans* frequently co-detected in these conditions (Li et al., 2023). A substantial body of *in vitro* and *in vivo* evidence confirms that these two species mutually enhance pathogenicity, forming robust dual-species biofilms with elevated cariogenic potential (Falsetta et al., 2014). While fluoride remains a cornerstone in caries prevention for ECC, its improper use can lead to dental fluorosis (Wong et al., 2024). For elderly patients with root caries, safer, naturally derived anti-cariogenic agents may offer a preferable alternative. Artemisinin is a well-established antimalarial with a proven clinical safety profile, yet its effects on cariogenic microorganisms have not previously been investigated. Moreover, anti-caries agents specifically designed to disrupt bacterial-fungal interactions remain limited.

This study is the first to investigate the inhibitory effects of seven artemisinin compounds on the synergistic cariogenesis of *C. albicans* and *S. mutans* both *in vitro* and *in vivo*. All seven compounds attenuated *C. albicans* virulence by inhibiting hyphal development, biofilm formation, and expression of *ALS3* and *EFG1*. And artemisinic acid and dihydroartemisinic acid showed broader activity, directly suppressing *S. mutans* growth at 400 mg/L and inhibiting its acidogenic and EPS-producing phenotypes. The other five compounds had little effect on *S. mutans* single-species biofilms but reduced *S. mutans* abundance, EPS production, and *gtfB/C* expression in dual-species biofilms at higher concentration. All *in vitro* phenotypic and transcriptional endpoints were evaluated after the same 24h treatment period in separate experiments, and their concordance represents only parallel, correlated responses, with no evidence of temporal or sequential causality.

Despite the lack of direct fungicidal activity, all compounds interfered with the pathogenicity of *C. albicans* by inhibiting hyphal development and biofilm formation. Hyphal growth is critical for *C. albicans* to adhere to dental surfaces, interact with *S. mutans*, and establish persistent biofilms. The downregulation of the key regulatory genes *ALS3* and *EFG1* provides a molecular correlate for this inhibitory effect (Sumlu et al., 2024). Als3 is an important determinant of *C. albicans* adhesion. It is highly expressed in strains causing oral mucosal infection, participates in adhesion to and aggregation with other microorganisms, and is a key regulator of *C. albicans* biofilm formation (Liu and Filler, 2011). Efg1 is a central regulator of hyphal growth in *C. albicans* (Xiong et al., 2021). The microscopy findings reinforce this interpretation: treated *C. albicans* biofilms contained fewer hyphae and predominantly yeast-form cells, while treated dual-species biofilms were less compact and contained less EPS. Thus, the compounds acted as anti-virulence agents against *C. albicans* even at concentrations that did not inhibit yeast form fungal growth. This distinction is relevant because weakening pathogenic traits without complete fungal eradication may be sufficient to reduce bacterial-fungal synergy. Our group previously work demonstrate the important contribution of *C. albicans* hyphae to the synergistic cariogenicity of *C. albicans-S. mutans*. All seven artemisinin compounds examined in the present study inhibited *C. albicans* hyphal formation, suggesting that interference with fungal morphogenesis may represent a shared mechanism through which these compounds attenuate dual-species cariogenicity. In addition to contributing to fungal adhesion and biofilm maturation, *C. albicans* hyphae may provide an expanded three-dimensional scaffold that facilitates the attachment and spatial organization of *S. mutans* within dual-species biofilms (Du et al., 2021; Liu et al., 2017; Liang et al., 2023). Consequently, inhibition of hyphal formation may weaken the physical basis of bacterial-fungal co-adhesion and limit the development of a densely organized, EPS-rich mixed community, even in the absence of direct bactericidal activity. This provides a plausible explanation for why compounds with limited effects on *S. mutans* single-species biofilms nevertheless reduced *S. mutans* abundance and EPS accumulation in dual-species biofilms.

Artemisinic acid and dihydroartemisinic acid exhibited unique dual activity. In addition to targeting *C. albicans*, they directly inhibited the growth of *S. mutans* at 400 mg/L, which distinguished them from the other five compounds and explained their greater reduction of single- and dual-species biofilm biomass. Their effects were consistently detected by crystal violet staining, CFU counting, SEM, and CLSM, reducing the likelihood that the observed antibiofilm activity reflected a single assay-specific phenomenon. The concentration-dependent responses across these complementary readouts further indicate that the two compounds affect both microbial accumulation and biofilm organization.

Beyond biofilm structure, this study evaluated two key processes in caries formation: acid production and EPS synthesis. Through glycolysis, *S. mutans* metabolizes sucrose into organic acids, principally lactic acid. Lactate dehydrogenase (LDH) is a key enzyme in lactic acid production by *S. mutans.* LDH reduces pyruvate to lactate, lowering the local pH at the tooth surface and ultimately causing dental demineralization (Xiong et al., 2020). The *ldh* gene is a key determinant of acid production in *S. mutans* (Qiu et al., 2017). Artemisinic acid and dihydroartemisinic acid inhibited acid production in both single- and dual-species biofilms, whereas the other compounds had no appreciable effect on acidogenicity. This was consistent with the finding that only artemisinic acid and dihydroartemisinic acid downregulated *ldh*. The agreement between pH measurements and gene-expression data suggests that suppression of acidogenic metabolism, in addition to reduced bacterial abundance, contributes to their anti-cariogenic effects. Nevertheless, because fewer viable cells can reduce their total acid output, future metabolic studies will be required to distinguish direct regulation of glycolysis from secondary effects of growth inhibition. EPS synthesis represents another pivotal factor in the synergistic interplay between *C. albicans* and *S. mutans*. *S. mutans* uses sucrose to synthesize glucan polymers and produces the glucosyltransferases GtfB, GtfC, and GtfD. Among the corresponding genes, *gtfB*/*C* play critical roles in the cariogenicity of *S. mutans* (Yamashita et al., 1993; Bowen and Koo, 2011). *C. albicans* produces relatively little EPS and adheres weakly to tooth surfaces (Matsui and Cvitkovitch, 2010; Gregoire et al., 2011).

However, in sucrose-rich environments, *S. mutans*-derived GtfB and GtfC synthesize extracellular glucans that assemble into an adhesive matrix on tooth surfaces and around bacterial microcolonies (Falsetta et al., 2014; Xiao et al., 2012). This EPS-rich matrix can surround both bacterial and fungal cells and enhance biofilm cohesion, thereby promoting co-adhesion between the two microorganisms and their attachment to tooth surfaces (Gregoire et al., 2011; Hwang et al., 2017). *C. albicans* can further enhance EPS synthesis by upregulating the expression of *gtf* genes in *S. mutans* (Cavalheiro and Teixeira, 2018; Kim et al., 2017). Previous studies have shown that deletion of *gtfB/C* markedly reduces the ability of *S. mutans* to form synergistic dual-species biofilms with *C. albicans* (Huffines and Scoffield, 2020; Ellepola et al., 2017). These findings indicate that Gtf-mediated glucan synthesis not only supports the intrinsic cariogenicity of *S. mutans* but also serves as a central link maintaining interspecies adhesion and synergistic cariogenicity between the two microorganisms. In untreated dual-species biofilms, *C. albicans* upregulated *gtfB/C* expression in *S. mutans*, consistent with increased EPS production, enhanced co-adhesion, and greater biofilm accumulation. Quantification of EPS using the sulfuric acid-anthrone assay, together with CLSM, confirmed that all seven artemisinin compounds reduced EPS production in dual-species biofilms. Artemisinic acid and dihydroartemisinic acid directly inhibited *gtfB/C* expression in *S. mutans* single-species biofilms, whereas the other compounds produced this effect only at 400 mg/L in dual-species biofilms. Given their lack of appreciable activity against *S. mutans* single-species biofilms and their ability to inhibit *C. albicans* hyphal growth, these compounds may act indirectly by disrupting fungal morphology and thereby weakening the stimulatory effect of *C. albicans* on bacterial glucan synthesis. Therefore, by effectively inhibiting *gtfB* and *gtfC* expression in *S. mutans*, artemisinic acid and dihydroartemisinic acid may further weaken the interaction between *C. albicans* and *S. mutans*, resulting in a greater reduction in their synergistic cariogenicity.

The anti-cariogenic efficacy of the artemisinin compounds was further supported by the rat caries models. Caries severity was substantially greater in rats co-infected with *C. albicans* and *S. mutans* than in those infected with *S. mutans* alone, whereas rats infected with *C. albicans* alone exhibited the mildest lesions and developed no Dx-level caries. All derivatives reduced *C. albicans* colonization in dental plaque, while only artemisinic acid and dihydroartemisinic acid significantly decreased *S. mutans* levels, consistent with the *in vitro* growth and single-species biofilm results. Keyes scoring showed that in the *C. albicans* infection group, all compounds reduced caries severity, suggesting that inhibition of *C. albicans* adhesion and hyphal development may attenuate its cariogenic potential. In the *S. mutans* infection group, only artemisinic acid and dihydroartemisinic acid reduced caries severity; the other treatment groups did not significantly differ from the negative control group. In rats co-infected with *C. albicans* and *S. mutans*, 400 mg/L artemisinic acid and dihydroartemisinic acid significantly reduced molar caries severity, with E-, Ds-, Dm-, and Dx-level lesion scores all differing significantly from those of the control group. Artemisinic acid and dihydroartemisinic acid were the most effective compounds in reducing the initiation of enamel-level lesions and preventing their progression into dentin, the other five compounds reduced only Dx-level lesion scores. These findings suggest that, even when the direct antimicrobial activity is limited, suppression of bacterial-fungal synergy can reduce caries severity.

Body-weight remained comparable across groups during the 5-weeks experiment, and no overt adverse effects were observed. These findings support preliminary *in vivo* tolerability but do not by themselves establish long-term or clinical safety. For eventual oral application, the effective concentrations, duration of exposure, local retention, effects on oral tissues, and effects on health-associated commensal microorganisms will need to be defined. Local delivery systems that maintain an effective concentration at plaque-retentive sites may be especially relevant, because the most significant effects of several compounds were observed at 200–400 mg/L. And comparative studies with fluoride and other established anti-caries agents should be needed to determine whether artemisinin compounds are best developed as alternatives, adjuncts, or components of combination formulations.

A potential advantage of naturally derived anticaries candidates is that they may act by attenuating pathogenicity rather than simply killing microorganisms, thereby potentially reducing selective pressure for resistance. In this study, artemisinic acid and dihydroartemisinic acid produced the most pronounced caries-preventive effects. The differences in activity among the derivatives also revealed a noteworthy structure-activity relationship. Artemisinic acid and dihydroartemisinic acid, which lack the peroxide bridge found in the other artemisinin compounds (Giacometti et al., 1996), nevertheless exhibited the strongest anticaries activity, suggesting that other structural moieties may contribute to their activity against cariogenic microorganisms. Therefore, future studies could identify the structural features responsible for the anticaries activity of artemisinin compounds and use structural optimization to design candidates which more effectively disrupt the synergistic cariogenicity of *C. albicans* and *S. mutans* for the prevention and treatment of ECC and root caries.

Several limitations should be considered. The controlled single- and dual-species models do not reproduce the taxonomic diversity, salivary components, dietary fluctuations, and spatial gradients present in dental plaque. More complex saliva-derived or defined multispecies biofilms should be used to determine whether the compounds retain activity in a competitive microbial community. The 5-week treatment period does not address long-term efficacy, microbial adaptation, pharmacokinetics, mucosal toxicity, or the possibility of resistance development. Testing in additional animal models and clinically relevant tooth models will be necessary before translation to human caries prevention. The present study did not separately quantify the water-soluble EPS fraction or assess *gtfD* expression. GtfD primarily synthesizes water-soluble glucans, which can serve as primers for GtfB-mediated glucan synthesis. Because GtfB is considered a major determinant of *S. mutans* cariogenicity, particularly through its contribution to adhesive water-insoluble glucan synthesis and biofilm matrix formation, we prioritized the measurement of water-insoluble EPS and *gtfB* expression (Huang et al., 2025; Wang et al., 2021). Therefore, future studies could separately quantify these EPS fractions and evaluate *gtfD* expression.

Future work should integrate structure-activity analysis with mechanistic and translational evaluation. Priorities include identifying the microbial targets of artemisinic acid and dihydroartemisinic acid, defining how the other compounds disrupt *C. albicans-S. mutans* interactions, optimizing local delivery and evaluating long-term effects in multispecies biofilms and animal models. These research could clarify whether the broad dual-target activity of artemisinic acid and dihydroartemisinic acid, or the more selective anti-virulence activity of the other derivatives.

## 5 Conclusion

**Figure 7:**
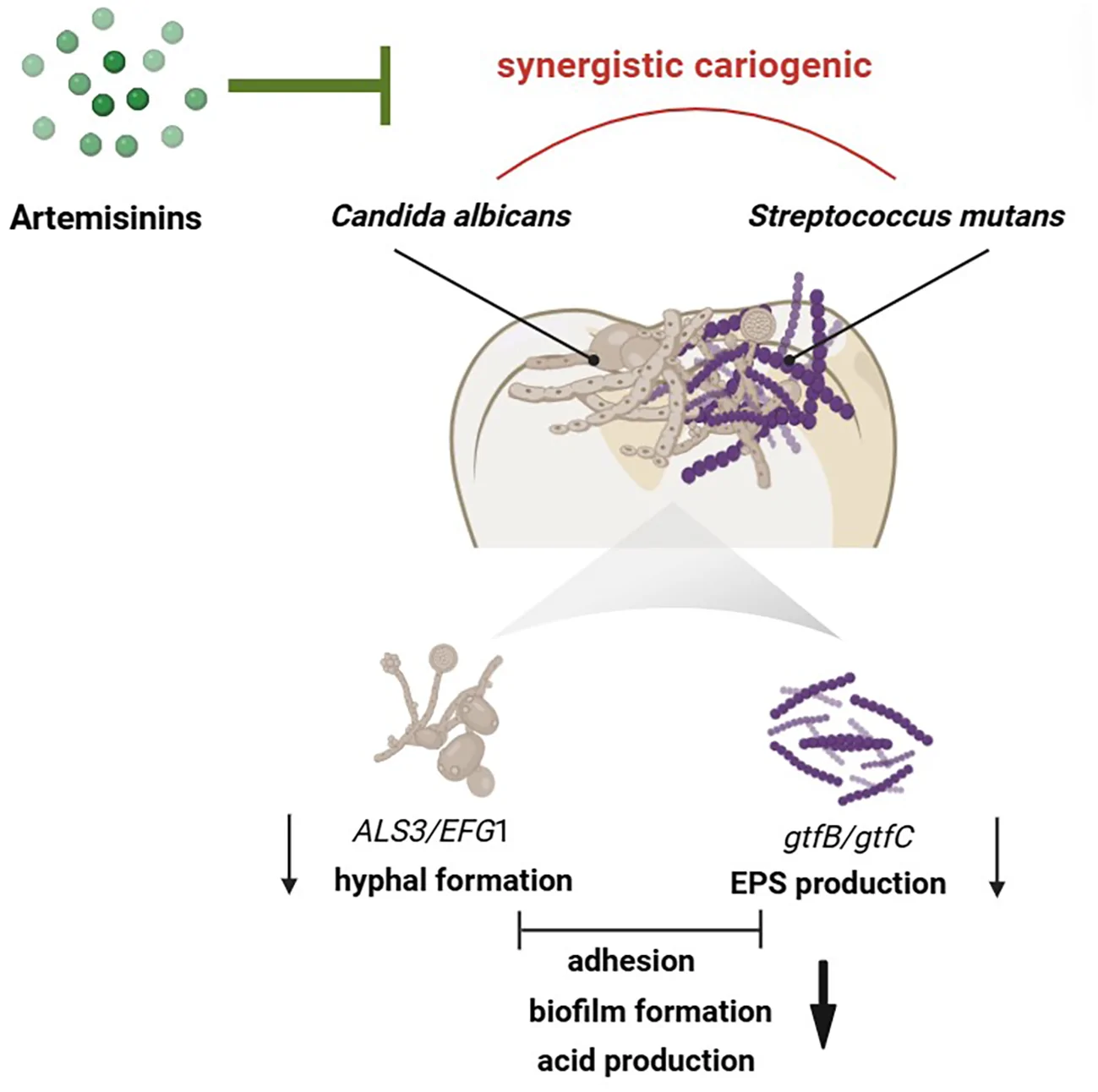
Schematic diagram of artemisinins inhibiting the synergistic cariogenicity of *C. albicans* and *S. mutans.*.

In this study, we demonstrated that artemisinin compounds attenuated the synergistic cariogenicity of *C. albicans* and *S. mutans* by inhibiting dual-species biofilm formation, fungal hyphal development, bacterial proliferation, acid production, and EPS synthesis (**Figure 7**). Artemisinic acid and dihydroartemisinic acid exhibited the broadest activity, targeting both microorganisms and multiple virulence processes, whereas the other five compounds primarily interfered with *C. albicans* pathogenicity and bacterial-fungal synergy. Collectively, these findings support the potential of artemisinin compounds as natural anticariogenic candidates for preventing and managing ECC and root caries. However, further structural optimization, mechanistic validation, long-term safety evaluation, and clinical investigation are required.

## Data Availability

The raw data supporting the conclusions of this article will be made available by the authors, without undue reservation.
