# Supplementary material for: Artemisinins inhibited the synergistic cariogenic effect between Candida albicans and Streptococcus mutans
Source: Front Cell Infect Microbiol. 2026 Sep 8;16:1950360. doi: 10.3389/fcimb.2026.1950360 (PMC13593465; doi:10.3389/fcimb.2026.1950360)
Supplement: Supplementary file 1 [file Table1.docx]

Supplementary Material

# 1 Supplementary Figures

**Supplementary Figure 1.** Artemisinin compounds inhibit hyphal development. Effects of the seven artemisinin compounds on hyphal formation by *C. albicans*.

**Supplementary Figure 2.** Changes in rat body weight during the experimental period. Body weights were monitored throughout the 5-week caries experiment for all infection and treatment groups. Values and statistical annotations are presented as defined in Materials and methods.

# 2 **Supplementary Tables**

| Primer | Sequence |
| --- | --- |
| *gtfB*-F  *gtfB*-R | 5’-AGCGTTGTCCGGATTTATTG-3’  5’-CTACGCATTTCACCGCTACA-3’ |
| *gtfC*-F  *gtfC*-F | 5’-CACTATCGGCGGTTACGAAT-3’  5’-CAATTTGGAGCAAGTCAGCA-3’ |
| *ldh*-F  *ldh*-R | 5’-GATGCTGCAAACTTCGAACA-3’  5’-TATTGACGCTGCGTTTCTTG-3’ |
| *ALS3*-F  *ALS3*-R | 5′-CCACTTCACAATCCCCATC-3′  5′-CAGCAGTAGTAGTAACAGTAGTAGTTTCATC-3′ |
| *EFG1*-F | 5’-TATGCCCCAGCAAACAACTG-3’ |
| *EFG1*-R | 5’-TTGTTGTCCTGCTGTCTGTC-3’ |

**Supplementary Table 1.** Primer sequences used for quantitative reverse-transcription PCR analysis. Forward and reverse primers are listed for the *S. mutans* virulence genes *gtfB*, *gtfC*, and *ldh*, the *C. albicans* hypha-associated genes *ALS3* and *EFG1*, and the corresponding reference genes.

| Compound | Lesion levels | | | |
| --- | --- | --- | --- | --- |
|  | E | Ds | Dm | Dx |
| Control | 36.0±1.0 | 29.2±1.2 | 17.0±2.5 | 5.3±1.6 |
| Artemisinic Acid | 29.3±1.9**** | 22.0±2.1**** | 13.8±2.4**** | 2.0±1.3**** |
| Dihydroartemisinic Acid | 28.0±1.3**** | 21.3±2.0**** | 12.5±2.5**** | 2.0±1.2**** |
| Artemisinin | 35.0±2.3 | 28.4±1.8 | 17.2±2.6 | 5.0±2.2 |
| Dihydroartemisinin | 35.3±2.2 | 28.6±2.4 | 16.5±2.3 | 4.8±1.9 |
| Artesunate | 36.3±1.2 | 29.0±1.4 | 17.0±2.4 | 4.9±2.1 |
| Artemether | 34.2±1.9 | 28.2±2.6 | 16.9±2.9 | 5.2±2.4 |
| Arteether | 34.4±2.0 | 27.1±1.6 | 16.2±2.7 | 4.7±2.0 |

**Supplementary Table 2.** Keyes scores for sulcal caries in rats infected with *S. mutans*. Scores are reported for enamel (E), slight dentinal (Ds), moderate dentinal (Dm), and extensive dentinal (Dx) lesions across the control and artemisinin compounds treatment groups.

| Compound | Lesion levels | | | |
| --- | --- | --- | --- | --- |
|  | E | Ds | Dm | Dx |
| Control | 18.2±2.0 | 12.9±1.9 | 7.5±1.5 | 0.0±0.0 |
| Artemisinic Acid | 15.6±2.2* | 10.0±2.0* | 2.2±1.3**** | 0.0±0.0 |
| Dihydroartemisinic Acid | 15.4±1.9* | 10.0±2.4 | 2.0±1.4**** | 0.0±0.0 |
| Artemisinin | 14.2±1.3** | 9.8±2.8* | 2.0±1.8**** | 0.0±0.0 |
| Dihydroartemisinin | 15.2±1.9* | 10.2±1.9 | 1.4±1.2**** | 0.0±0.0 |
| Artesunate | 14.0±2.2** | 8.9±1.2** | 2.1±1.5**** | 0.0±0.0 |
| Artemether | 14.7±1.5** | 9.2±1.3** | 2.0±1.2**** | 0.0±0.0 |
| Arteether | 12.5±2.3**** | 9.2±2.0** | 1.6±0.9**** | 0.0±0.0 |

**Supplementary Table 3.** Keyes scores for sulcal caries in rats infected with *C. albicans*. Scores are reported for enamel (E), slight dentinal (Ds), moderate dentinal (Dm), and extensive dentinal (Dx) lesions across the control and artemisinin compounds treatment groups.

| Compound | Lesion levels | | | |
| --- | --- | --- | --- | --- |
|  | E | Ds | Dm | Dx |
| Control | 42.3±2.5 | 36.8±2.9 | 26.5±2.2 | 11.2±2.0 |
| Artemisinic Acid | 35.7±2.3**** | 30.0±2.6**** | 16.3±2.4**** | 4.3±1.9**** |
| Dihydroartemisinic Acid | 33.5±1.4**** | 28.0±1.4**** | 16.8±2.3**** | 2.8±2.0**** |
| Artemisinin | 41.3±1.5 | 36.0±3.0 | 25.0±1.3 | 8.3±1.0* |
| Dihydroartemisinin | 41.3±2.4 | 35.6±2.3 | 24.8±2.3 | 8.2±2.7* |
| Artesunate | 41.8±2.6 | 36.0±1.4 | 25.3±1.3 | 8.0±1.7* |
| Artemether | 40.0±1.8 | 35.3±2.2 | 24.5±2.4 | 7.8±2.4* |
| Arteether | 41.5±1.9 | 35.5±2.7 | 23.8±2.5 | 8.0±2.0* |

**Supplementary Table 4.** Keyes scores for sulcal caries in rats co-infected with *S. mutans* and *C. albicans*. Scores are reported for enamel (E), slight dentinal (Ds), moderate dentinal (Dm), and extensive dentinal (Dx) lesions across the control and artemisinin compounds treatment groups.
